# Tumors Established in a Defective Immune Environment Reprogram the Oncogenic Signaling Pathways to Escalate Tumor Antigenicity

**DOI:** 10.3390/biomedicines12040846

**Published:** 2024-04-11

**Authors:** Chiao-Hsu Ke, Hsin-Yi Wu, Yu-Shan Wang, Wei-Hsiang Huang, Chen-Si Lin

**Affiliations:** 1 Department of Veterinary Medicine, School of Veterinary Medicine, National Taiwan University, Taipei 10617, Taiwan; f08629002@ntu.edu.tw (C.-H.K.); d91629002@ntu.edu.tw (Y.-S.W.); 2 Instrumentation Center, National Taiwan University, Taipei 10617, Taiwan; hsinyiwu@ntu.edu.tw; 3 Uni-Pharma Co., Ltd., Taipei 11494, Taiwan; 4 Graduate Institute of Molecular and Comparative Pathobiology, School of Veterinary Medicine, National Taiwan University, Taipei 10617, Taiwan; whhuang@ntu.edu.tw

**Keywords:** tumor suppressors, immunogenicity, tumor antigens, proliferative cell signaling, PTEN

## Abstract

Tumors developed in immunocompromised hosts are more immunogenic. However, few studies have addressed the potential mechanisms underlying the high immunogenicity of tumors found in a suppressed immune system. Therefore, we aimed to elucidate the impacts of the immune system on tumor behaviors and immunogenicity sculpting. A murine colorectal adenocarcinoma cell line, CT26wt, was administrated into immunocompetent (BALB/c) and immunocompromised (NOD.SCID) mice, respectively. On day 11, the CT26 cells slowly progressed in the NOD.SCID mice compared to the BALB/c mice. We then performed liquid chromatography–tandem mass spectrometry (LC-MS/MS) and analyzed the differentially expressed proteins (DEPs). The DEPs participated in numerous oncogenic pathways, PI3K/AKT/mTOR cell signaling, and the silencing of several tumor suppressors, such as PTEN and RBL1, during tumorigenesis. On day 34, the CT26/SCID tumors inversely became malignant counterparts; then the CT26/SCID tumors were harvested and re-inoculated into immunocompetent mice (CT26/SCID-Re tumors) to determine the immunogenicity. The CT26/SCID-Re tumor growth rate significantly decreased. Furthermore, increased infiltrations of dendritic cells and tumor-infiltrating T lymphocytes were found in the CT26/SCID-Re tumors. These findings suggest that immunogenic tumors might express multiple tumor rejection antigens, unlike wild-type tumors, and attract more immune cells, therefore decreasing the growth rate. Collectively, our study first revealed that in immunodeficient hosts, tumor suppressors were silenced and oncogenic signaling pathways were changed during the initial phase of tumor development. With tumor progression, the tumor antigens were overexpressed, exhibiting elevated immunogenicity. This study offers a hint on the mechanisms of tumorigenesis and provides a niche for investigating the interaction between host immunity and cancer development.

## 1. Introduction

Tumor cells are usually transformed from normal cells, and these cells require genetic mutations in proto-oncogenes and/or tumor suppressors [1]. With the dysregulation of tumor-associated genes, tumors aberrantly develop and release danger signals that activate the immune system. When antigen-presenting cells, especially dendritic cells (DCs), sense and capture tumor cells, the DCs undergo maturation, migrate to the draining lymph nodes (dLNs), and deliver tumor antigens into major histocompatibility complex (MHC) class I molecules for presentation to CD8+ T cells [2]. These lead to the activation of antigen-specific CD8+ T cells, which are known as cytotoxic T lymphocytes (CTLs). CTLs can identify the antigenic targets expressed on the tumors and secrete IFN-γ, perforin, and granzyme, thus triggering antitumor responses [3,4]. In other words, tumor rejection antigens, including tumor-associated antigens (TAAs) and tumor-specific antigens (TSAs), are the targets of antitumor CTLs [5]. However, tumors decrease their antigenicity to escape from CTLs by downregulating these tumor rejection antigens. The above description highlights the significance of retaining highly immunogenic antigens expressed on the tumor cells in the design of personalized immunotherapy.

To survive immunosurveillance, tumors create a favorable tumor microenvironment (TME) and escape from host immunity through various mechanisms. Within the TME, the dysfunction of the immune system allows tumors to escape immune detection by reducing tumor antigens and their ability to provoke an immune response. These include the formation of regulatory immune cells [6], production of suppressive cytokines [7], and diminished expression of tumor rejection antigens [8]. Certain regulatory immune cells, including regulatory T cells (Tregs), myeloid-derived suppressive cells (MDSCs), dysfunctional DCs, and M2 macrophages, produce factors like indoleamine 2,3-dioxygenase (IDO) and arginase to create an immunosuppressive environment [9]. Tumors also release cytokines that suppress the immune function and trigger effector T cells to transform into Tregs by secreting IL-10 and TGF-β [10]. These Tregs then compete with the effector T cells by largely consuming IL-2, a vital cytokine for T cell activation and maintenance [11,12]. Furthermore, Tregs secret perforin and granzyme to kill the CTLs, leading to osmotic lysis and apoptosis [6]. Tregs can also produce IL-10, TGF-β, and IL-35 to dampen antitumor immunity, for example, by the suppression of antigen presentation by DCs, and decrease the antitumor abilities of CTLs [13]. Tumors downregulate their expressed antigens and the MHC complex, which the immune system can recognize, in order to evade immunosurveillance. When tumors are established in immunodeficient hosts, without immune recognition, they can progressively outgrow those developing in immunocompetent individuals [14,15]. With the consistent instability within tumors, the dysregulation of cell signaling, which manipulates cell proliferation, facilitates tumor development. The uncontrolled growth in tumors ultimately results in alterations of tumor antigens, some of which might have high levels of immunogenicity [16].

Tumors will change their traits within an immunocompromised host, constructing an environment that facilitates tumor progression and thereby increasing tumor immunogenicity [14,15]. For example, tumors originating from immunodeficient (RAG2^−/−^) mice were more capable of triggering an immune response, indicating that these tumors were more immunogenic than those in wild-type (WT) mice [17]. Escors et al. suggested that RAG2^−/−^x γc^−/−^ mice were more sensitive to MCA (3-methylcholanthrene)-induced sarcoma than syngeneic RAG2^−/−^ and WT mice [16]. Furthermore, an exome sequence analysis revealed that tumors from RAG2^−/−^ mice, unlike those from WT mice, displayed elevated immunogenicity by determining the mutational landscape, in which a point mutation in Spectrin-β2, a neo-epitope, was identified [18]. Consistent with these publications, our previous study demonstrated similar findings: tumors arising in immunocompromised, i.e., NOD.CB17-Prkdcscid/NcrCrl (NOD.SCID), mice exhibited more malignant phenotypes and increased immunogenicity [14]. These results suggested that tumors derived from a defective immune system expressed a higher level of immunogenic tumor antigens. However, the molecular characteristics of tumors developed in these hosts have rarely been reported. Few studies have compared the unfamiliar/foreign TMEs created by NOD.SCID mice in comfortable or stressed environments. Furthermore, the mechanisms by which tumors develop in NOD.SCID mice that express more immunogenic antigens remain to be elucidated. Therefore, in the current study, we aimed to clarify the impacts of a defective TME on tumors and provide direct evidence that tumors express elevated immunogenic antigens within immunocompromised hosts.

## 2. Materials and Methods

### 2.1. Animals and Cells

All studies were conducted with approval by the Institutional Animal Care and Use Committee, National Taiwan University (IACUC No. NTU109-EL-00135). Female six- to eight-week-old BALB/c and NOD.SCID mice were housed in pathogen-free conditions. The murine colon carcinoma cell line CT26.WT was obtained from the America Type Culture Collection (ATCC^®^ No. CRL-2638™). The culture medium and supplements were RPMI-1640 (Thermo Fisher Scientific, Waltham, MA, USA, Catalog No. 11875093), 10% FBS (Thermo Fisher Scientific, Catalog No. 10100147), and 1% antibiotic-antimycotic (AA) (GeneDireX, Taipei, Taiwan, Catalog No. CC501-0100). CT26.WT cells were cultured in a humidified incubator with 5% CO_2_ at 37 °C and routinely screened for *Mycoplasma* (Merck, Darmstadt, Germany, Catalog No. MP0040A). 

The CT26.WT cells (3 × 10^5^/mouse) were subcutaneously administrated in the dorsal ileum regions of mice. Mice were withdrawn from the study due to reaching the end of experiment, having ulcerations at the tumor site, or having tumor volumes above 1500 mm^3^. For survival analysis, mortality was based on mouse removal from the study once the tumor was above 1500 mm^3^ or ulcerations were observed at tumor sites. For the animal studies, tumor volumes were measured every three days. Tumor volumes were calculated as width^2^ × length × 0.5 using calipers. 

### 2.2. Tumor Harvesting, Digestion, and Re-Inoculation

To evaluate the immunogenicity of tumors from CT26-tumor-bearing mice, the tumors were collected for further analysis. Necrotic areas were excluded, and the regions of tumors were harvested for further analysis. For tumor digestion, the single-tumor-cell digestion protocol was followed as previously described with simple modification [19]. Briefly, tumors ranging in size from 500 to 1000 mm^3^ were manually cut into pieces measuring 3–5 mm using a surgical scalpel. Then, the dissected tumors were further chopped into 100 µm segments using maximum blade force with a Mcllwain tissue chopper. These tumor samples were then immersed in 500 µL of 10× collagenase–hyaluronidase solution, containing 150,000 U of collagenase (Sigma-Aldrich, St. Louis, MO, USA, Catalog No. C9891) and 5000 U of hyaluronidase (Sigma-Aldrich, Catalog No. H3506) dissolved in MEM and Hanks’ balanced salts (Gibco, Grand Island, NY, USA, Catalog No. 12571063) dissolved in 4.5 mL high-glucose DMEM (Gibco, Catalog No. 11995-073) containing 5% FBS at 37 °C and shaken at 200 rpm for 30 min. Samples were disrupted by pipetting them up and down every 10 min to ensure the tissues were sufficiently resuspended. Then samples were washed by adding 10 mL of cold DPBS (Gibco, Catalog No. 14190-250) to neutralize digestion and centrifugated at 200× *g* for 5 min at 4 °C. After disposal of the supernatant, the pellets were resuspended in 100 µL of DNase solution (Roche, Mannheim, Germany, Catalog No. 10104159001) and mixed with 750 µL TEG (10 mL of 2.5% trypsin, Gibco, Catalog No. 15090-046; 40 mg of EGTA, Sigma-Aldrich, Catalog No. E3889; 10 mg of polyvinyl alcohol, Sigma-Aldrich, Catalog No. P8136; 90 mL of DPBS) by pipetting them up and down for 1 min at 37 °C. Then 15 mL DPBS containing 2% FBS was added to the samples, and they were centrifugated at 200× *g* for 5 min at 4 °C. The supernatant was aspired, and 1.25 mL of Dipase solution (Sigma-Aldrich, Catalog No. SCM133) was added before incubation for 5 min in a 37 °C water bath. The cell pellets were resuspended in 50 µL DNase solution and 450 µL DPBS containing 2% FBS. Red blood cells were lysed with 2 mL RBC lysis buffer (Sigma-Aldrich, Catalog No. R7757) and incubated for 2 min at 37 °C in a water bath. Samples were washed with 15 mL DPBS containing 2% FBS and spun at 200 g for 5 min at 4 °C. Following the removal of the supernatant, the pellets were resuspended with 10 mL of cold DPBS containing 2% FBS and filtered through a 40 µm cell sterile strainer (Falcon, Durham, NC, USA, Catalog No. 352340). The resulting cells were collected, washed twice with serum-free DPBS, and then resuspended in DPBS for tumor inoculation into BALB/c mice. 

BALB/c mice were subcutaneously injected with CT26 cells derived from BALB/c or NOD.SCID mice (1 × 10^6^/mouse) into the dorsal ileum regions. The exclusion criteria, survival analysis, and tumor volume measurements were the same as previously described. Tumor-free mice were defined as having a tumor mass below 9 mm in average diameter at day 14 [20]. 

### 2.3. Protein Preparation and LC-MS/MS Analysis

CT26/BALB/c and CT26/SCID tumors were harvested and immediately stored in liquid nitrogen to analyze the total protein expression profile. All the frozen tissues were pulverized with a mortar and pestle, and liquid nitrogen with RIPA lysis buffer (Sigma-Aldrich, Catalog No. R0278) supplemented with protease inhibitors (Thermo Fisher Scientific, Catalog No. 78425) and phosphatase inhibitors (Thermo Fisher Scientific, Catalog No. 78420) was used to extract the total proteins. The samples were collected and kept on ice. Lysates were then centrifuged for 20 min at 16,000× *g* at 4 °C, and insoluble debris was discarded. The supernatant proteins were precipitated with a 4-fold volume of pre-cooled acetone and incubated for 1 h at −80 °C. The resulting samples were centrifuged at 14,000× *g* for 10 min. The precipitated proteins were dissolved in 6 M urea, and the total protein concentration was measured with a BCA kit (Bio-Rad Laboratories, Hercules, CA, USA). A total of 50 µg of proteins was reduced at 29 °C by 5 mM DTT (dithiothreitol) for 45 min and then alkylated with 10 mM iodoacetamide in the dark at 29 °C for 45 min. The proteins were digested by trypsin (Promega, Madison, WI, USA; V5111) with a 50:1 (*w*/*w*) ratio at 29 °C for 16 hr. The reaction was quenched by adding 10% trifluoroacetic acid (TFA) to the final concentration of 0.5% TFA. The final solution was desalted using the C_18_ procedure [14]. 

Liquid chromatography–tandem mass spectrometry (LC-MS/MS) analysis was conducted on an Orbitrap Fusion Lumos Tribrid quadrupole ion trap–Orbitrap mass spectrometer (Thermo Fisher Scientific). A C_18_ Acclaim PepMap NanoLC column (2.0 μm × 75 μm × 250 mm) (Thermo Fisher Scientific) on an Ultimate System 3000 nanoLC system (Thermo Fisher Scientific) was used to separate the peptides. Mobile phase A was H_2_O with 0.1% formic acid (FA), while mobile phase B was acetonitrile (CAN) with 0.1% FA. Following each injection, the peptides were eluted at a gradient of 2% to 40% for 90 min with a flow rate of 300 ng/mL. A full MS scan was performed and followed by high-energy collision-activated dissociation (HCD)-MS/MS of the most intense ions in 3 secs. External calibration for mass accuracy was <5 ppm. The resolution for MS1 was set to 120,000 at *m*/*z* 200, and a target value of 5 × 10^5^ ions for both scan types was chosen. The maximum ion injection time was set to 50 msec for the full scan. HCD-MS/MS, with the resolution set to 15,000, was used to fragment multiply charged ions within a 1.4 Da isolation window at a normalized collision energy of 32 MS/MS (AGC target 5 × 10^4^; maximum injection time of 50 msec) with previously selected ions dynamically excluded for 180 s. 

The MS/MS spectra were searched against the mouse sequence database (17131 entries released in November 2022) using the Mascot search engine (Matrix Science, London, UK; version 2.3) and Sequest by Proteome Discoverer 2.5. The parameters specified for label-free quantification were as follows: Trypsin was specified for the enzymatic digestion with two missed cleavages and precursor mass tolerance of 10 ppm; and fragment mass error tolerance of 0.02 Da was allowed. Deamidation (NQ) and oxidation (M) were set as variable modifications and carbamidomethylation (C) was set as a fixed modification. To minimize false positives, a peptide mass tolerance with a false detection rate (FDR) of <1% was used for accurate protein observation [21]. To identify differentially expressed proteins (DEPs), protein abundances above 2-fold change were defined as overexpressed proteins, and those below 0.5-fold change were defined as underexpressed proteins [22]. 

### 2.4. Bioinformatic Analysis

Identified DEPs were imported into Database Annotation Visualization and Integrated Discovery (DAVID, http://david.abcc.ncifcrf.gov/, accessed on 19 January 2024) to investigate the Gene Ontology (GO) terms, including biological processes (BPs), cellular components (CCs), and molecular functions (MFs) [23], and the enriched Kyoto Encyclopedia of Genes and Genomes (KEGG) pathways [24]. Reactome (http://reactome.org, accessed on 19 January 2024) was employed to determine the enriched biomolecular pathways that DEPs involved [25]. To compare the expression patterns of DEPs, Morpheus (https://software.broadinstitute.org/morpheus/, accessed on 19 January 2024) was applied to generate clustered heat map [26]. The Search Tool for the Retrieval of Interacting Genes/Proteins (STRING, https://string-db.org/, accessed on 19 January 2024) network was employed to establish a protein–protein interaction (PPI) network of DEPs [27]. 

### 2.5. Immunofluorescence Staining and Analysis

To determine the infiltrations of immune cells, an immunofluorescence staining assay was employed. The blocks were sectioned at 5 μm and deparaffinized. Then the sections underwent antigen retrieval in boiling citrate buffer (at pH of 6.0) for 12 min. A fluorescence multiple-stain kit (BioTnA, Kaohsiung, Taiwan) was applied for the immunofluorescence staining. To block non-specific bindings, the Hi-effect Immunoblock blocking buffer was employed for incubation for 2 h at room temperature. Slides were incubated with primary antibodies: CD3 (Abcam, Cambridge, UK, clone SP162, Catalog No. ab135372, dilution factor: 1:200), CD11c (Abcam, clone EP1347Y, Catalog No. ab52632, dilution factor: 1:200), and CD103 (Abcam, clone EPR22590-27, Catalog No. ab224202, dilution factor: 1:500). After the incubation of primary antibodies, the slides were washed and separately incubated with the secondary antibodies for 30 min at room temperature. The corresponding secondary antibodies were goat anti-rabbit IgG-FAM, goat anti-rabbit IgG-TRMRA, and goat anti-rabbit IgG-Cy5. After the last step, the DNA binding dye, DAPI, was applied to the slides and incubated for 1 min, followed by a 5 min wash [14]. Each sample was independently counted in five random microscopic fields at 40× objective magnification (0.1 mm^2^). The slides were separately analyzed by two veterinary pathologists who were blinded to the experimental story. Sections in necrotic areas or located in the margins of the tissues were excluded from consideration to avoid artifacts [28]. 

### 2.6. Statistical Analysis

The data are presented as mean ± standard derivation (SD). Statistical analyses were performed in GraphPad Prism v9 (GraphPad Software, La Jolla, CA, USA). To determine significant differences, two-way ANOVA with Tukey multiple comparisons, Student’s *t* test, and nonparametric Mann–Whitney U test were utilized. Differences in survival curves were compared by log-rank (Mantel–Cox) test with correction for multiple pair-wise comparisons. Differences were regarded as statistically significant at a *p*-value of less than 0.05 (*, *p* < 0.05; **, *p* < 0.01; ***, *p* < 0.001; ****, *p* < 0.0001). 

## 3. Results

### 3.1. Tumors Developed in Immunocompromised Hosts Had a Decreased Growth Rate

**Figure 1:**
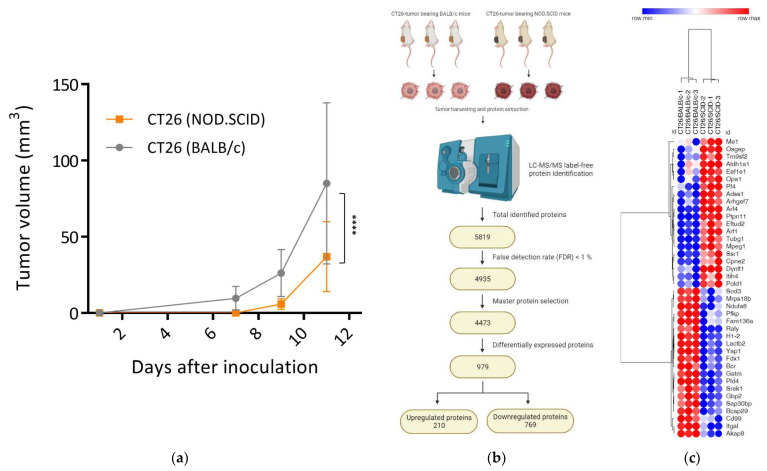
Different tumor growth and protein expression profiles in tumors developed in NOD.SCID mice during tumorigenesis. (**a**) The CT26 tumors were injected into NOD.SCID and syngeneic BALB/c mice, and tumor growth rates are shown. (**b**) Schema of proteomic analysis of CT26 tumors from the NOD.SCID and BALB/c mice. CT26 tumor masses of NOD.SCID and BALB/c mice were harvested after they were sacrificed, and the total proteins were extracted for LC/MS.MS analysis. A total of 5819 proteins were identified, and 4935 proteins with an FDR of <1% were validated. Followed by the selection of master protein, 4473 proteins were verified for further analysis. After selection, 979 DEPs were filtered, including 219 upregulated proteins and 769 downregulated proteins. (**c**) Distinct protein expression profiles of tumors developed in BALB/c and SCID mice during the tumor initiation. Heatmap of representative DEPs between CT26/BALC/c and CT26/SCID tumors was generated using Morpheus networks by hierarchical clustering. The protein abundance is shown from high (red) to low (blue). LC/MS.MS, liquid chromatography–tandem mass spectrometry; FDR, false detection rate; DEPs, differentially expressed proteins. ****, *p* < 0.0001.

Palpable tumors were found in all of the BALB/c mice (n = 10), whereas tumors failed to generate in all of the NOD.SCID mice (n = 10) on day seven after the injection. On day 11, a rapid tumor growth pattern was found in the BALB/c mice, with sizes reaching 85.01 ± 52.81 mm^3^, as compared to 36.95 ± 22.93 mm^3^ in the NOD.SCID mice (*p* < 0.001, Figure 1a). To investigate the mechanisms of tumor progression, we harvested the tumors derived from these two strains of mice and conducted an LC-MS/MS analysis. A total of 979 proteins were identified as DEPs, including 769 downregulated proteins and 210 upregulated proteins (Figure 1b). The inconsistency of DEPs in the CT26/SCID and CT26/BALB/c tumors demonstrated that the proteomic profiles were significantly changed (Figure 1c), and alterations in protein expression were revealed to be the reason behind the slow development of CT26/SCID.

### 3.2. Silence of Tumor Suppressors and Reprogramming of Proliferative Signaling Pathways in CT26/SCID Tumors

**Figure 2:**
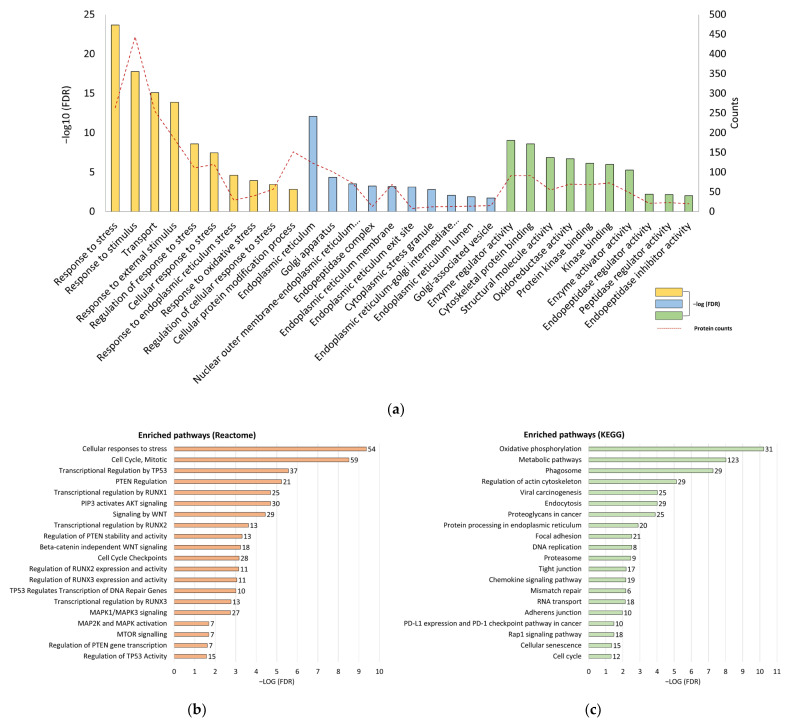
Enriched GO terms, Reactome, and KEGG pathways the DEPs involved. Stressed CT26 tumors developed in NOD.SCID mice induce the silence of tumor suppressors and the dysfunction of ER. (**a**) Representative enriched GO terms, including BP, CC, and MF, and the (**b**) enriched Reactome (**c**) and KEGG pathways in the DEPs. The color bar represents -log (FDR), and the dotted red line (GO terms) and number next to the bars show the protein numbers involved in each signal pathway. GO, Gene Ontology; KEGG, Kyoto Encyclopedia of Genes and Genomes; DEPs, differentially expressed proteins; BP, biological process; CC, cellular component; MF, molecular function; FDR, false detection rate.

Functional analyses, including GO terms and an enriched signaling pathway analysis, were employed to uncover the interaction among the DEPs. Most of the enriched GO terms were related to cell stress, endoplasmic reticulum (ER) stress, and endopeptidase regulation, including “regulation of cellular response to stress”, “oxidative stress”, and “response to endoplasmic reticulum stress” (Figure 2a). Enriched Reactome pathways were related to the downregulation of tumor suppressors and cell growth signaling pathways, such as “transcriptional regulation by TP53”, “PTEN regulation”, “PIP3 activates AKT signaling”, and “mTOR signaling” (Figure 2b). Enriched KEGG pathways were involved in “protein processing in endoplasmic reticulum”, “DNA replication”, and “RAP1 signaling pathways” (Figure 2c). Numerous well-known tumor suppressors, including PTEN and RBL1, were downregulated or absent in CT26/SCID tumors (Table S1). These findings might suggest that tumors developing in a stressed/foreign environment (without an intact immune system) inhibited the tumor suppressors and changed the cellular growth signals in response to surviving in the stressed environment.

### 3.3. CT26/SCID Tumors Progressively Developed at the Endpoint

The aforementioned findings suggested the downregulation of tumor suppressors during CT26/SCID tumorigenesis. To analyze whether these behaviors remained during tumor progression, we observed the tumor growth rate and assessed the protein expression profiles on day 34. In contrast to the growth patterns at tumor initiation, CT26 tumors inversely grew faster in the NOD.SCID mice, with tumor sizes reaching 2049.90 ± 1050.65 mm^3^, as compared with those in the BALB/c mice, which had sizes of 1366.81 ± 454.38 mm^3^ on day 21 (*p* < 0.05, Figure S1a). The NOD.SCID mice displayed a shorter overall survival time after tumor inoculation (*p* < 0.05, Figure S1b). Our previous study uncovered the total protein expression profiles [14], which indicated that CT26/SCID tumors express numerous immunogenic tumor antigens. Taken together, these results suggest that the decrease in tumor suppressors in the early stage facilitates tumor progression and gene instability, which promote the immunogenic tumor antigens overexpressed in CT26/SCID tumors. 

### 3.4. Highly Immunogenic CT26/SCID Tumors Trigger Robust Adaptive Immune Responses

**Figure 3:**
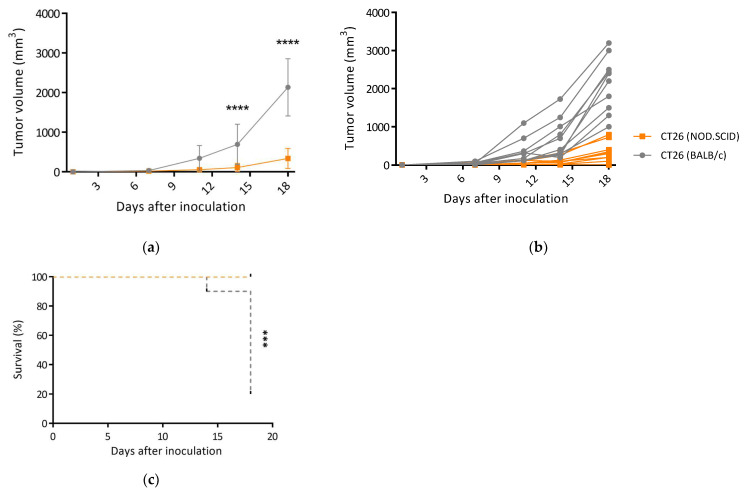
A majority of tumors derived from NOD.SCID mice fail to re-form when transplanted into syngeneic WT mice. CT26-derived cell lines were derived from tumors generated in NOD.SCID and BALB/c (WT) mice. These cell lines were transplanted into syngeneic BALB/c hosts, and tumor growth and survival were measured over time. (**a**) Average and (**b**) individual tumor growth curves are shown (n = 10 in each group). (**c**) Survival of mice transplanted with CT26-derived cell lines prepared from NOD.SCID and BALB/c mice (n = 10). For survival analysis, mortality was based on the number of mice removed from the study once the tumor was above 1500 mm^3^ or the mice that had ulcerations at tumor sites. Data are representative of 3–4 independent experiments. Bar graphs reflect mean ± SD (n = 10) analyzed by two-way ANOVA with Tukey’s post-hoc test or Kaplan–Meier survival method for survival analysis. ***, *p* < 0.001; ****, *p* < 0.0001.

The tumors from the two strains of mice were harvested to determine their immunogenicity. Following single-cell digestion, the CT26/BALB/c and CT26/SCID tumor cells were separately re-inoculated into the BALB/c mice to evaluate their ability to trigger adaptive immune responses. When these cell lines were transplanted into WT mice, the CT26/BALB/c tumor cells formed progressively growing tumor masses (CT26/BALB/c-Re) by days 14 and 18 (*p* < 0.0001, Figure 3a). A total of 70% of the CT26/SCID tumors re-inoculated into the BALB/c mice were regressors (CT26/SCID-Re). In contrast, only one CT26/BALB/c tumor regressed, and 90% of these tumors were progressors (Figure 3b). Therefore, the CT26/BALB/c-Re tumor-bearing mice exhibited decreased overall survival after tumor inoculation (*p* < 0.001, Figure 3c). These results suggest a higher editing level of the immune system in the BALB/c mice versus the NOD.SCID mice, in which the “unedited” tumors triggered more robust antitumor abilities.

**Figure 4:**
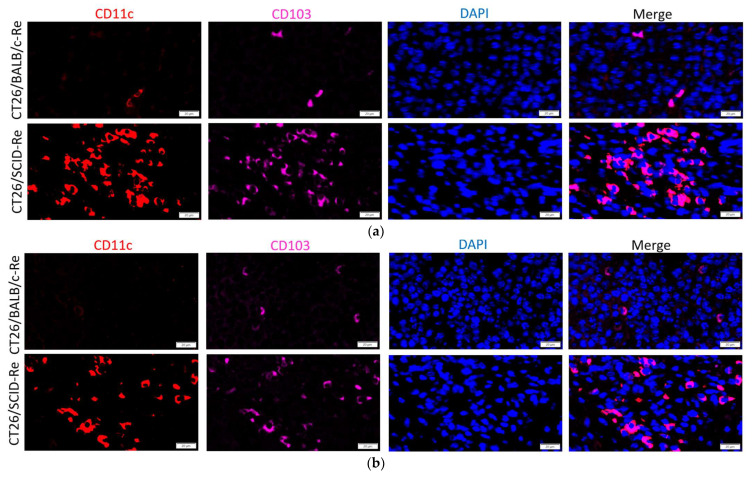

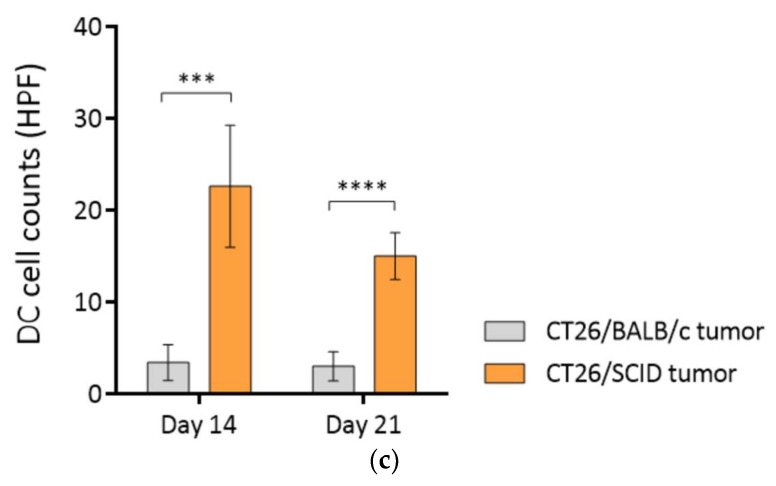
CT26/SCID tumors are more immunogenic with the increased attraction of CD103+CD11c+ DCs. (**a**) Representative immunofluorescence staining showing CT26/BALB/c and CT26/SCID tumors infiltrated by CD103+CD11c+ DCs on day 14; (**b**) Representative immunofluorescence staining showing CT26/BALB/c and CT26/SCID tumors infiltrated by CD103+CD11c+ DCs on day 21; (**c**) Statistical analysis of DC infiltrations on days 14 and 21 within tumors. Data are representative of 3–4 independent experiments. Bar graphs reflect mean ± SD. Statistical analyses were performed with the unpaired Student’s *t* test. ***, *p* < 0.001; ****, *p* < 0.0001; HPF, high-power field.

**Figure 5:**
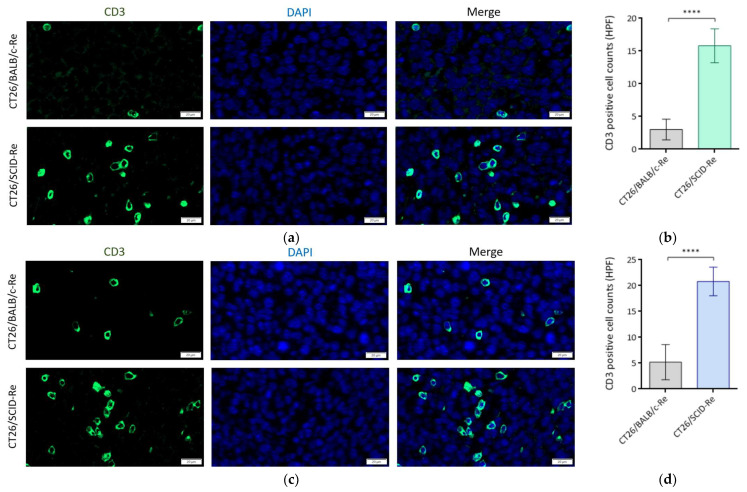
Tumor regressors are edited when transplanted into BALB/c mice with the increased recruitment of tumor-infiltrating lymphocytes. (**a**) Representative immunofluorescence staining and (**b**) statistical analysis show CT26/BALB/c and CT26/SCID tumors infiltrated by CD3+ T cells on day 14; (**c**) Representative immunofluorescence staining and (**d**) statistical analysis show CT26/BALB/c and CT26/SCID tumors infiltrated by CD3+ T cells on day 21. Data are representative of 3–4 independent experiments. Bar graphs reflect mean ± SD. Statistical analyses were performed with the unpaired Student’s *t* test. ****, *p* < 0.0001; HPF, high-power field.

Then, the tumors were harvested for further immune analysis. The CT26/SCID-Re tumors attracted more CD11c+CD103+ DCs than the CT26/BALB/c tumors on day 14 (Figure 4a) and day 21 (Figure 4b), which indicated that the CT26/SCID tumors harbored more immunogenic antigens and thus increased the infiltrations of antigen-presenting cells (Figure 4c). Furthermore, on day 14, the amounts of CD3+ T cells within the CT26/SCID-Re tumors were significantly higher than those in CT26/BALB/c tumors (Figure 5a,b). Similar findings were also found on day 21 (Figure 5c,d). These results echoed the tumor growth rates, wherein the tumors with increased their antigenicity first, being attracted the infiltrations of DCs. Then, after T cell education, the CTLs destroyed the tumors, leading to the high regression rate of the CT26 tumor cells derived from the NOD.SCID mice (Figure 3a,b). 

## 4. Discussion

The loss of function of tumor suppressors plays a critical role in tumor development [29,30]. The deletion of tumor suppressors facilitates tumor progression, thereby keeping these tumor cells in a continuous proliferative state [31,32]. Our results showed that numerous tumor suppressors were downregulated in the CT26/SCID tumors (Table S1), which promoted cell proliferation signaling (Figure 2b); however, the tumor growth rate remained slow (Figure 1a). As shown in Figure 1c, an inconsistency of the DEPs in the CT26/SCID and CT26/BALB/c tumors was found. For example, PTEN [33], RBL1 [34], CDKN2AIP [35], RNASET2A [36], HINT1 [37], and PARP12 [38] are well-known tumor suppressors and were downregulated or absent in the CT26/SCID tumors (Table S1). The enriched signaling pathways involved in cell cycles, namely, PIP3-activated AKT signaling, mTOR signaling, WNT signaling, and MAPK activation, suggested that the manipulation of tumor growth was significantly changed in the NOD.SCID mice. Notably, *PTEN*, one of the most commonly somatically mutated or deleted genes in cancer [33], was absent in the CT26/SCID tumors. Such an absence directly activates PI3K signaling and thus triggers the downstream activation of the AKT protein [39]. Phospho-AKT-activated mTOR regulates cell growth, survival, and proliferation [40]. While the dysregulation of *PTEN* disturbs PI3K/AKT/mTOR signaling, its inactivation can lead to gene instability and thus induce the formation of tumor neoantigens [30]. Therefore, in the current study, we presumed that the CT26 tumors developed in the NOD.SCID mice induced the downregulation of tumor suppressors and thus facilitated tumor progression by manipulating their cell cycles and/or cell growth signaling pathways. However, these changes did not influence the present tumor growth pattern (day 11) but had further impacts on tumor development and antigen shaping (day 34). On day 34, with malignant development (Figure S1a), the tumors expressed numerous TAAs [14], which are byproducts of tumor development, as a result of the loss of function of tumor suppressors such as *PTEN* in the CT26/SCID tumors. On the other hand, several tumor-related oncogenic pathways in the CT26/SCID tumors were enriched. These suggest that tumor patients with low immune status might have opportunities to control their tumor’s size by targeting these signaling pathways. However, more evidence is needed to support this hypothesis. 

The tumors developed in the NOD.SCID mice might have undergone cell stress due to the lower tumor growth rate on day 11 (Figure 1a) and the enriched biological pathways involved in “cell stress” and “ER stress” (Figure 2a). The CT26 tumor cells originated in the BALB/c mice; in the development of the NOD.SCID mice, the TME created by the NOD.SCID mice was significantly different from those in parental environments. A foreign environment, such as the dysregulation of growth signals (Figure 2b,c) and loss of tumor suppressors (Table S1) [41], can expose tumor cells to stress, pose challenges for protein processing in the ER [42], and therefore cause ER stress [43]. Factors that can evoke ER stress in tumors facilitate tumor cell growth and cancer progression in response to surviving the foreign environment [44]. Thus, CT26/SCID tumor cells might undergo ER stress and thereby promote tumor development (Figure 3a). However, it would be prudent to verify these findings in the future. 

Consistent with previous studies, we found that tumors arising in immunodeficient (NOD.SCID) mice are more immunogenic [16,17]. The findings of the current study could uncover further potential mechanisms for this. The tumors developed in the NOD.SCID mice result in the loss of tumor suppressors, thereby generating neoantigens [30]. Furthermore, tumor cells with ER stress can increase their immunogenicity [45]. A previous study also reported that stressed cells induce the generation of antigenic peptides [46]. The results from this study strengthen our aforementioned findings. The regressor frequency of the CT26 tumor cells derived from the NOD.SCID mice (CT26/SCID-Re tumors) was 70% (7/10). Moreover, almost all (9/10) of the CT26 tumor cells derived from BALB/c successfully developed in three independent experiments (Figure 3a,b). Because a majority of CT26/SCID-Re tumors are regressors, we speculate that the primary tumor cell repertoire consisted of mostly immunogenic tumor cells that were immunologically heterogeneous [20]. This heterogenicity can be partially or fully sculpted by the complete immune system in BALB/c mice, and therefore, immunogenic tumor antigens were downregulated. We identified numerous TAAs in the CT26/SCID tumors, and without immune systems, the immunogenic antigens were retained, which could activate robust immune responses in immunocompetent individuals (Figure 4 and Figure 5). 

In the current study, we found that tumors grown in immunocompromised mice were more immunogenic by changing biological pathways and downregulating tumor suppressor genes. Therefore, based on these findings, we can provide a simple and convenient platform to improve the immunogenicity of autologous cancer vaccines (ACVs). Many clinical studies have shown that ACVs have poor levels of immunogenicity [9]. Therefore, in the future, it might be possible to collect some clinical specimens and inject them into NOD.SCID mice and then enhance the immunogenicity of tumors to treat cancer. In fact, our previous publication showed that these highly immunogenic ACVs can provoke strong immune responses in a rodent model [14]. Therefore, combined with a detailed mechanism analysis from the current study, we expect that this immunotherapy can be translated into clinical medicine in the future. 

This study had some limitations. Though we comprehensively analyzed the total protein profiles using LC-MS/MS to minimize bias, the lack of other cell lines currently restricts further exploration. Further studies using other cell lines for comparison and validation are needed. Second, the detailed mechanisms for the slower tumor growth rate in NOD.SCID mice should be elucidated to strengthen our findings. Lastly, we proved the high tumor antigenicity of CT26/SCID-Re by re-inoculating tumors and analyzing the DCs and TILs (Figure 4 and Figure 5). Further biological experiments, such as ones in which CT26/SCID-Re tumor cells are co-cultured with DCs and/or T cells to analyze their cytokine production, mRNA expression, and antitumor functions, are highly recommended. 

## 5. Conclusions

In conclusion, this study has reported the traits of tumors derived from immunocompromised hosts. To survive a stressed environment, CT26/SCID tumor cells negatively regulated tumor suppressors, facilitating tumor progression. With their malignant development, the TAAs, the byproducts of tumor growth, were overexposed. This study demonstrated the increased tumor immunogenicity of CT26/SCID tumors and reported the detailed mechanisms underlying tumor development in NOD.SCID mice. Finally, we revealed the elevated immunogenicity of tumors developed in NOD.SCID mice by showing that CT26/SCID-Re tumors are reproducible and correlate with the level of immune cells in the tumor-bearing hosts. Our study provides an effective way to increase the immunogenicity of tumors with solid evidence-based medicine. We hope to apply this concept to clinical medicine in the future.

## Data Availability

The data presented in this study are available upon request from the corresponding author.

## Supplementary Materials

The following supporting information can be downloaded at: https://www.mdpi.com/article/10.3390/biomedicines12040846/s1, Figure S1: CT26/SCID tumors exhibiting malignant phenotypes; Table S1: Differentially expressed protein identified from LC-MS/MS analysis of CT26 tumors (NOD.SCID mice) Vs CT26 tumors (BALB/c mice).

## References

[1] Sun W., Yang J. (2010). Functional mechanisms for human tumor suppressors. J. Cancer.

[2] Roberts E.W., Broz M.L., Binnewies M., Headley M.B., Nelson A.E., Wolf D.M., Kaisho T., Bogunovic D., Bhardwaj N., Krummel M.F. (2016). Critical Role for CD103(+)/CD141(+) Dendritic Cells Bearing CCR7 for Tumor Antigen Trafficking and Priming of T Cell Immunity in Melanoma. Cancer Cell.

[3] Kaech S.M., Cui W. (2012). Transcriptional control of effector and memory CD8+ T cell differentiation. Nat. Rev. Immunol..

[4] Nolz J.C. (2015). Molecular mechanisms of CD8(+) T cell trafficking and localization. Cell. Mol. Life Sci..

[5] Jhunjhunwala S., Hammer C., Delamarre L. (2021). Antigen presentation in cancer: Insights into tumour immunogenicity and immune evasion. Nat. Rev. Cancer.

[6] Li C., Jiang P., Wei S., Xu X., Wang J. (2020). Regulatory T cells in tumor microenvironment: New mechanisms, potential therapeutic strategies and future prospects. Mol. Cancer.

[7] Berraondo P., Sanmamed M.F., Ochoa M.C., Etxeberria I., Aznar M.A., Pérez-Gracia J.L., Rodríguez-Ruiz M.E., Ponz-Sarvise M., Castañón E., Melero I. (2019). Cytokines in clinical cancer immunotherapy. Br. J. Cancer.

[8] Garcia-Lora A., Algarra I., Garrido F. (2003). MHC class I antigens, immune surveillance, and tumor immune escape. J. Cell Physiol..

[9] Ke C.-H., Chiu Y.-H., Huang K.-C., Lin C.-S. (2023). Exposure of Immunogenic Tumor Antigens in Surrendered Immunity and the Significance of Autologous Tumor Cell-Based Vaccination in Precision Medicine. Int. J. Mol. Sci..

[10] Paluskievicz C.M., Cao X., Abdi R., Zheng P., Liu Y., Bromberg J.S. (2019). T Regulatory Cells and Priming the Suppressive Tumor Microenvironment. Front. Immunol..

[11] Spolski R., Li P., Leonard W.J. (2018). Biology and regulation of IL-2: From molecular mechanisms to human therapy. Nat. Rev. Immunol..

[12] Carmenate T., Ortíz Y., Enamorado M., García-Martínez K., Avellanet J., Moreno E., Graça L., León K. (2018). Blocking IL-2 Signal In Vivo with an IL-2 Antagonist Reduces Tumor Growth through the Control of Regulatory T Cells. J. Immunol..

[13] Sullivan J.A., Tomita Y., Jankowska-Gan E., Lema D.A., Arvedson M.P., Nair A., Bracamonte-Baran W., Zhou Y., Meyer K.K., Zhong W. (2020). Treg-Cell-Derived IL-35-Coated Extracellular Vesicles Promote Infectious Tolerance. Cell Rep..

[14] Ke C.-H., Wang Y.-S., Chiang H.-C., Wu H.-Y., Liu W.-J., Huang C.-C., Huang Y.-C., Lin C.-S. (2022). Xenograft cancer vaccines prepared from immunodeficient mice increase tumor antigen diversity and host T cell efficiency against colorectal cancers. Cancer Lett..

[15] Ke C.-H., Tomiyasu H., Lin Y.-L., Huang W.-H., Huang H.-H., Chiang H.-C., Lin C.-S. (2022). Canine transmissible venereal tumour established in immunodeficient mice reprograms the gene expression profiles associated with a favourable tumour microenvironment to enable cancer malignancy. BMC Vet. Res..

[16] Escors D. (2014). Tumour Immunogenicity, Antigen Presentation, and Immunological Barriers in Cancer Immunotherapy. New J. Sci..

[17] Schreiber R.D., Old L.J., Smyth M.J. (2011). Cancer immunoediting: Integrating immunity’s roles in cancer suppression and promotion. Science.

[18] Matsushita H., Vesely M.D., Koboldt D.C., Rickert C.G., Uppaluri R., Magrini V.J., Arthur C.D., White J.M., Chen Y.S., Shea L.K. (2012). Cancer exome analysis reveals a T-cell-dependent mechanism of cancer immunoediting. Nature.

[19] Rodriguez de la Fuente L., Law A.M.K., Gallego-Ortega D., Valdes-Mora F. (2021). Tumor dissociation of highly viable cell suspensions for single-cell omic analyses in mouse models of breast cancer. STAR Protoc..

[20] O’Sullivan T., Saddawi-Konefka R., Vermi W., Koebel C.M., Arthur C., White J.M., Uppaluri R., Andrews D.M., Ngiow S.F., Teng M.W. (2012). Cancer immunoediting by the innate immune system in the absence of adaptive immunity. J. Exp. Med..

[21] Madda R., Chen C.-M., Wang J.-Y., Chen C.-F., Chao K.-Y., Yang Y.-M., Wu H.-Y., Chen W.-M., Wu P.-K. (2020). Proteomic profiling and identification of significant markers from high-grade osteosarcoma after cryotherapy and irradiation. Sci. Rep..

[22] Zhou J., Liu B., Li Z., Li Y., Chen X., Ma Y., Yan S., Yang X., Zhong L., Wu N. (2021). Proteomic Analyses Identify Differentially Expressed Proteins and Pathways Between Low-Risk and High-Risk Subtypes of Early-Stage Lung Adenocarcinoma and Their Prognostic Impacts. Mol. Cell. Proteom..

[23] Gene Ontology C. (2006). The Gene Ontology (GO) project in 2006. Nucleic Acids Res..

[24] Dennis G., Sherman B.T., Hosack D.A., Yang J., Gao W., Lane H.C., Lempicki R.A. (2003). DAVID: Database for Annotation, Visualization, and Integrated Discovery. Genome Biol..

[25] Fabregat A., Sidiropoulos K., Viteri G., Forner O., Marin-Garcia P., Arnau V., D’Eustachio P., Stein L., Hermjakob H. (2017). Reactome pathway analysis: A high-performance in-memory approach. BMC Bioinform..

[26] Ryan M.C., Stucky M., Wakefield C., Melott J.M., Akbani R., Weinstein J.N., Broom B.M. (2019). Interactive Clustered Heat Map Builder: An easy web-based tool for creating sophisticated clustered heat maps. F1000Research.

[27] Franceschini A., Szklarczyk D., Frankild S., Kuhn M., Simonovic M., Roth A., Lin J., Minguez P., Bork P., von Mering C. (2013). STRING v9.1: Protein-protein interaction networks, with increased coverage and integration. Nucleic Acids Res..

[28] Ke C.-H., Sio K.-M., Wang S.-L., Kuo Y., Huang W.-H., Lin C.-S. (2022). The High Expression of Legumain in Canine Neoplasms: A Retrospective Analysis of 100 Cases. Animals.

[29] Wang P., Zhang H., Yang J., Li Z., Wang Y., Leng X., Ganapathy S., Isakson P., Chen C., Zhu T. (2020). Mu-KRAS attenuates Hippo signaling pathway through PKCι to sustain the growth of pancreatic cancer. J. Cell. Physiol..

[30] Vidotto T., Melo C.M., Castelli E., Koti M., Dos Reis R.B., Squire J.A. (2020). Emerging role of PTEN loss in evasion of the immune response to tumours. Br. J. Cancer.

[31] Wellenstein M.D., de Visser K.E. (2018). Cancer-Cell-Intrinsic Mechanisms Shaping the Tumor Immune Landscape. Immunity.

[32] Rooney M.S., Shukla S.A., Wu C.J., Getz G., Hacohen N. (2015). Molecular and genetic properties of tumors associated with local immune cytolytic activity. Cell.

[33] Lee Y.R., Chen M., Pandolfi P.P. (2018). The functions and regulation of the PTEN tumour suppressor: New modes and prospects. Nat. Rev. Mol. Cell Biol..

[34] Naert T., Dimitrakopoulou D., Tulkens D., Demuynck S., Carron M., Noelanders R., Eeckhout L., Van Isterdael G., Deforce D., Vanhove C. (2020). RBL1 (p107) functions as tumor suppressor in glioblastoma and small-cell pancreatic neuroendocrine carcinoma in Xenopus tropicalis. Oncogene.

[35] Cao Y., Chen Z., Qin Z., Qian K., Liu T., Zhang Y. (2022). CDKN2AIP-induced cell senescence and apoptosis of testicular seminoma are associated with CARM1 and eIF4β. Acta Biochim. Biophys. Sin..

[36] Bruno A., Noonan D.M., Valli R., Porta G., Taramelli R., Mortara L., Acquati F. (2022). Human RNASET2: A Highly Pleiotropic and Evolutionary Conserved Tumor Suppressor Gene Involved in the Control of Ovarian Cancer Pathogenesis. Int. J. Mol. Sci..

[37] Li H., Balajee A.S., Su T., Cen B., Hei T.K., Weinstein I.B. (2008). The HINT1 tumor suppressor regulates both gamma-H2AX and ATM in response to DNA damage. J. Cell Biol..

[38] Shao C., Qiu Y., Liu J., Feng H., Shen S., Saiyin H., Yu W., Wei Y., Yu L., Su W. (2018). PARP12 (ARTD12) suppresses hepatocellular carcinoma metastasis through interacting with FHL2 and regulating its stability. Cell Death Dis..

[39] Maehama T., Dixon J.E. (1998). The tumor suppressor, PTEN/MMAC1, dephosphorylates the lipid second messenger, phosphatidylinositol 3,4,5-trisphosphate. J. Biol. Chem..

[40] Lee J.O., Yang H., Georgescu M.M., Di Cristofano A., Maehama T., Shi Y., Dixon J.E., Pandolfi P., Pavletich N.P. (1999). Crystal structure of the PTEN tumor suppressor: Implications for its phosphoinositide phosphatase activity and membrane association. Cell.

[41] Ozcan U., Ozcan L., Yilmaz E., Düvel K., Sahin M., Manning B.D., Hotamisligil G.S. (2008). Loss of the tuberous sclerosis complex tumor suppressors triggers the unfolded protein response to regulate insulin signaling and apoptosis. Mol. Cell.

[42] Oakes S.A. (2020). Endoplasmic Reticulum Stress Signaling in Cancer Cells. Am. J. Pathol..

[43] Dejeans N., Manié S., Hetz C., Bard F., Hupp T., Agostinis P., Samali A., Chevet E. (2014). Addicted to secrete—Novel concepts and targets in cancer therapy. Trends Mol. Med..

[44] Mahadevan N.R., Rodvold J., Sepulveda H., Rossi S., Drew A.F., Zanetti M. (2011). Transmission of endoplasmic reticulum stress and pro-inflammation from tumor cells to myeloid cells. Proc. Natl. Acad. Sci. USA.

[45] Lee S.Y., Oh J.Y., Kang T.H., Shin H.S., Cheng M.A., Farmer E., Wu T.C., Hung C.F. (2019). Endoplasmic reticulum stress enhances the antigen-specific T cell immune responses and therapeutic antitumor effects generated by therapeutic HPV vaccines. J. Biomed. Sci..

[46] Preynat-Seauve O., Coudurier S., Favier A., Marche P.N., Villiers C. (2003). Oxidative stress impairs intracellular events involved in antigen processing and presentation to T cells. Cell Stress Chaperones.

