# Supplementary material for: Tumors Established in a Defective Immune Environment Reprogram the Oncogenic Signaling Pathways to Escalate Tumor Antigenicity
Source: Biomedicines. 2024 Apr 11;12(4):846. doi: 10.3390/biomedicines12040846 (PMC11047836; doi:10.3390/biomedicines12040846)
Supplement: Supplementary file 1 [file biomedicines-12-00846-s001.zip › Supplementary figure.pdf]

## Supplementary figure

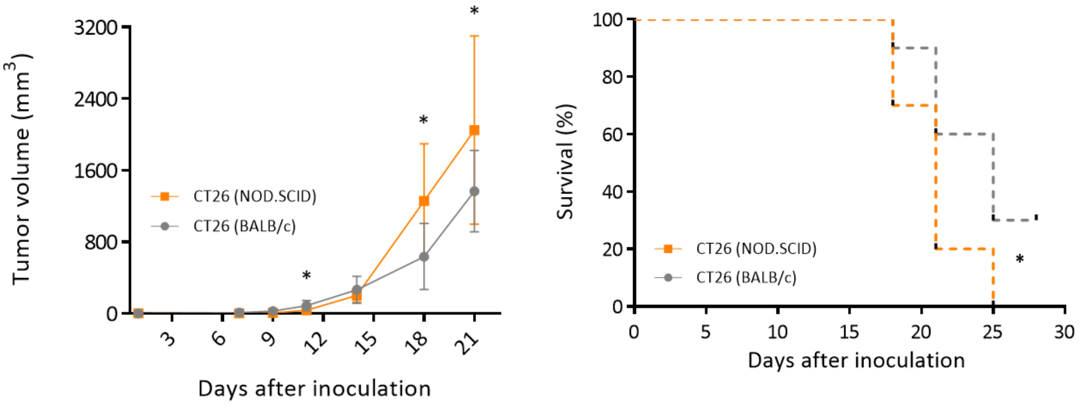


(**a**) (**b**)

**Figure S1.** CT26/SCID tumors exhibiting malignant phenotypes. (**a**) The CT26 tumors were injected into NOD.SCID and syngeneic BALB/c mice. The tumor volume from each strain was (n = 10); (**b**) Overall survival time was evaluated and mice were excluded from this study due to ulcerations at the tumor site or tumor volumes above 1500 mm$^3$. Statistical analyses were performed with two-way ANOVA and Tukey's post hoc test, Kaplan–Meier survival method for survival analysis, or Mann–Whitney U test. n.s., no significant difference; *, $P < 0.05$; **, $P < 0.01$.
